# Vagus Nerve Stimulation Reduces Body Weight and Fat Mass in Rats

**DOI:** 10.1371/journal.pone.0044813

**Published:** 2012-09-28

**Authors:** Sebastiano Banni, Gianfranca Carta, Elisabetta Murru, Lina Cordeddu, Elena Giordano, Francesco Marrosu, Monica Puligheddu, Gabriele Floris, Gino Paolo Asuni, Angela Letizia Cappai, Silvia Deriu, Paolo Follesa

**Affiliations:** 1 Department of Biomedical Sciences, University of Cagliari, Cagliari, Italy; 2 Department of Public Health and Clinical and Molecular Medicine, University of Cagliari, Cagliari, Italy; 3 Department of Life and Environmental Sciences, and Center of Excellence for the Neurobiology of Dependence, University of Cagliari, Cagliari, Italy; University of Santiago de Compostela School of Medicine - CIMUS, Spain

## Abstract

Among the manifold effects of vagus nerve stimulation (VNS) delivered as an add-on treatment to patients with drug-resistant epilepsy, a moderate loss of body weight has been observed in some individuals. We have now investigated this effect in rats. Exposure of rats to VNS for 4 weeks reduced feed conversion efficiency as well as body weight gain (by ∼25%) and the amount of mesenteric adipose tissue (by ∼45%) in comparison with those in sham-operated control animals. A pair-fed experiment showed that both lower dietary intake and increase energy expenditure independently contributed to the reduction of body weight and mesenteric adipose tissue. Moreover, VNS increased the level of non-esterified fatty acids in plasma and mesenteric adipose tissue by ∼50 and 80%, respectively, without affecting that in the liver. In addition, VNS reduced the amounts of endocannabinoids and increased *N*-palmitoylethanolamide, an endogenous ligand of the transcription factor PPARα (peroxisome proliferator–activated receptor α) in mesenteric adipose tissue but not in the hypothalamus. These effects were accompanied by increased expression of the gene for brain-derived neurotrophic factor (BDNF) in the hypothalamus and up-regulation of the abundance of PPARα in the liver. Our results suggest that the reduction in body fat induced by VNS in rats may result from the action of both central and peripheral mediators. The reduced feed conversion efficiency associated with VNS may be mediated by hypothalamic BDNF, down-regulation of endocannabinoid tone in mesenteric adipose tissue and a PPARα-dependent increase in fatty acid oxidation in the liver, which in concerted action may account for the anorexic effect and increased energy expenditure.

## Introduction

Long-term vagus nerve stimulation (VNS), characterized by intermittent delivery of a low-intensity electric current to the vagus nerve, has been introduced as an alternative add-on treatment for epilepsy. Experience with this approach worldwide indicates that 40 to 50% of individuals with drug-resistant epilepsy show a >40% reduction in the number of seizures and that it is well tolerated, with only a few transitory side effects [1]. Among such side effects, cough and hoarseness during the on-phase of stimulation occur relatively frequently during the first 6 months to 1 year, whereas other effects, such as dyspnea and throat pain, develop in ∼20% of patients during the 2^nd^ year, and nausea and vomiting may be experienced by some subjects at any time [1]–[3]. A small proportion of treated individuals has also been found to experience weight loss [2], [4]–[6], an effect that appears not to be related either to the antiepileptic action of VNS or to interaction between the prosthetic device and pharmacological treatments [6]. Although such weight loss has been proposed to be directly related to various telemetric settled VNS parameters or to result from peculiar individual sensitivity [5], the mechanism responsible for this “side” effect has remained as yet largely unknown. Direct electrical stimulation of vagus nerve branches at the thoracic or abdominal level in rats [7] or pigs [8], was found to result in weight loss, suggesting a possible role for mesenteric tissues targeted by the vagus nerve in inducing this effect. It is thus possible that, as in these cases stimulating directly the vagus nerve at thoracic or abdominal level, weight loss is achieved directly as a result of altered peristalsis/reduced absorption of nutrients. On the other hand, no data are so far available on whether stimulating the central nervous system by afferent fibers of the vagus nerve at the cervical level, as utilized for the antiepileptic action of VNS, the effect could be achieved indirectly by the combination of a top-down central effect on metabolism via reduced feed conversion efficiency.

We have previously shown that acute or chronic VNS elicits an increased expression of neurotrophic factors, including that of the neurotrophin brain-derived neurotrophic factor (BDNF), in various regions of the brain in rats [9], [10]. Neurotrophins are a family of growth factors that exert many of their effects on neurons through Trk receptor tyrosine kinases. Among neurotrophins and their receptors, BDNF and its receptor TrkB are the most widely and abundantly expressed in the brain and regulate neuronal development and synaptic plasticity [11]. BDNF has also been identified as a key component of pathways that controls body weight and energy homeostasis [12], [13], as was first suggested by the observation that intracerebroventricular infusion of BDNF in mice with genetic obesity due to a leptin receptor mutation results in reduced food intake, body weight, and blood glucose levels [14]. Moreover, obesity-related phenotypes have been described in heterozygous BDNF knockout mice as well as in mice in which the BDNF gene has been deleted selectively in excitatory neurons in the brain [15]. Thus, BDNF seems to be involved in regulating key aspects of both metabolism and energy balance. However, the mechanism by which BDNF alters energy balance and the relation of TrkB signaling to the major pathways previously shown to mediate the regulation of energy balance remains as yet unknown.

Other factors that may play a role in the effects of VNS are represented by the endocannabinoids and by the transcription factor peroxisome proliferator–activated receptor α (PPARα) and its endogenous ligands *N*-palmitoylethanolamide (PEA) and *N*-oleoylethanolamide (OEA) [16], [17]. Endocannabinoids are a group of neuromodulatory lipids that interact with cannabinoid receptors and thereby modulate various aspects of physiology of appetite [18], [19]. Injection of exogenous cannabinoids into the hypothalamus has been shown to activate a pathway responsible for food-seeking behavior [20]. Peripheral mechanisms may also contribute to the control of body weight by endocannabinoids. Indeed adipocytes are implicated in the control of endocannabinoid availability, affecting energy balance via central orexigenic drive and peripheral lipogenesis [21]–[23]. The endogenous ligands for PPARα are metabolically related to endocannabinoids and have been shown to inhibit food intake, reduce body weight gain, and stimulate lipolysis and fatty acid utilization [16], [17].

We have now examined the effects of VNS on feed conversion efficiency, body weight, and adipose tissue metabolism in rats. We thus measured the effects of chronic VNS on the tissue levels of non-esterified fatty acids (NEFA), central and peripheral endocannabinoid levels, central BDNF gene expression, and the hepatic abundance of PPARα.

## Methods

### Ethical Approval

All animal procedures were performed in accordance with the European Communities Council Directive of 24 November 1986 (86/609/EEC), including adequate measures to minimize pain or discomfort. The experimental protocols were also approved by the Animal Ethics Committee of the University of Cagliari.

**Figure 1:**
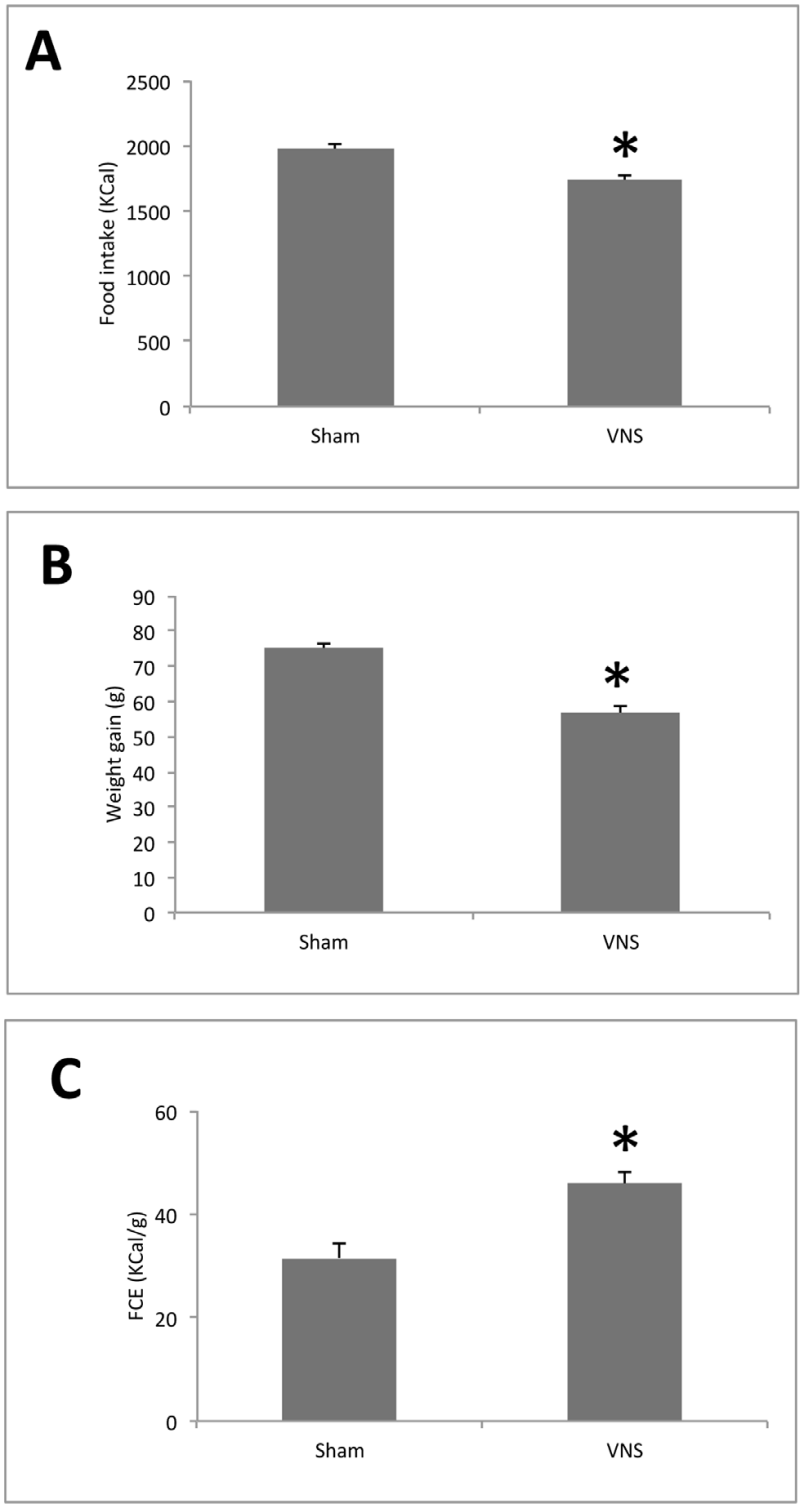
Effects of chronic VNS on food intake, gain in body weight and feed conversion efficiency (FCE) in rats. (**A**) Change in food intake (**B**) Change in body weight over 4 weeks. (**C**) FCE was determined from the number of kilocalories consumed per gram of weight gain. All data are means ± SEM for nine animals per group. *P<0.05 versus the corresponding value for sham-operated controls (Scheffe’s test).

**Figure 2:**
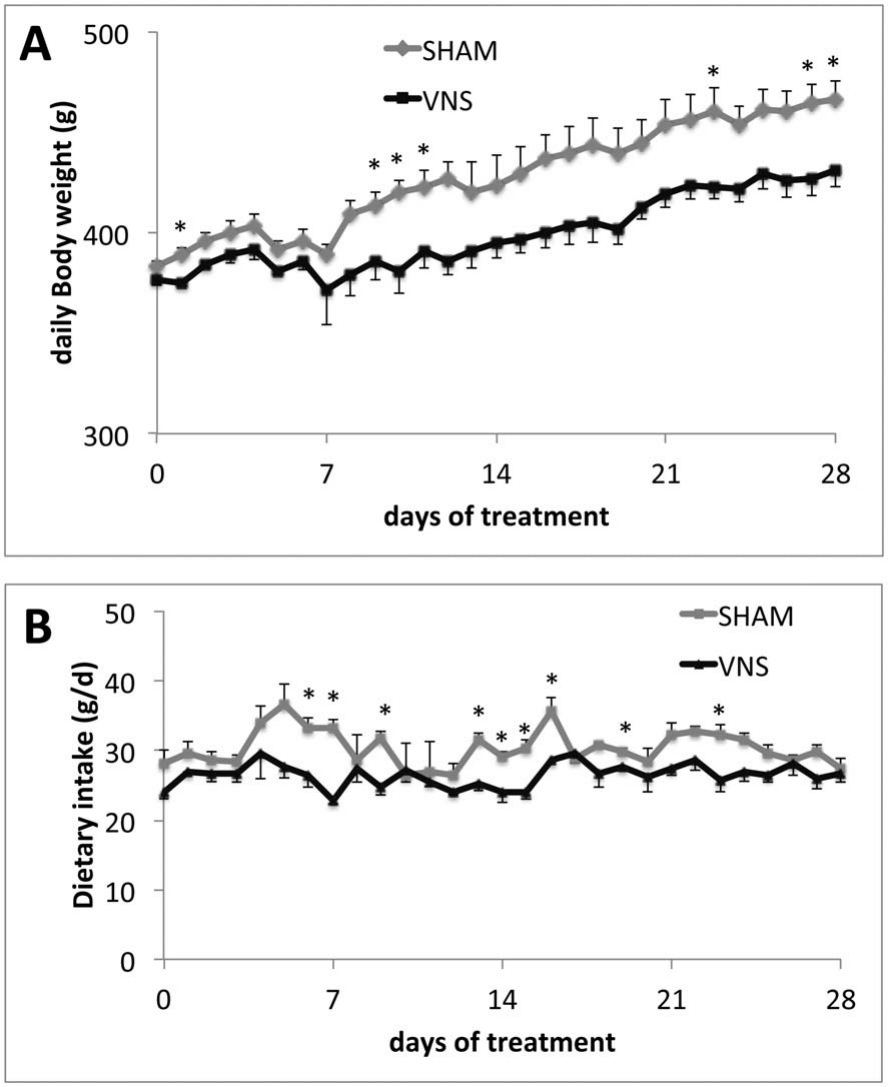
Effect of chronic VNS on daily body weight and daily food intake during 4 week-treatment. (A) body weight; (B) daily food intake. Data are means ± SEM for nine animals for each experimental group. *P<0.05 versus the value for sham-operated controls (*t* test).

**Figure 3:**
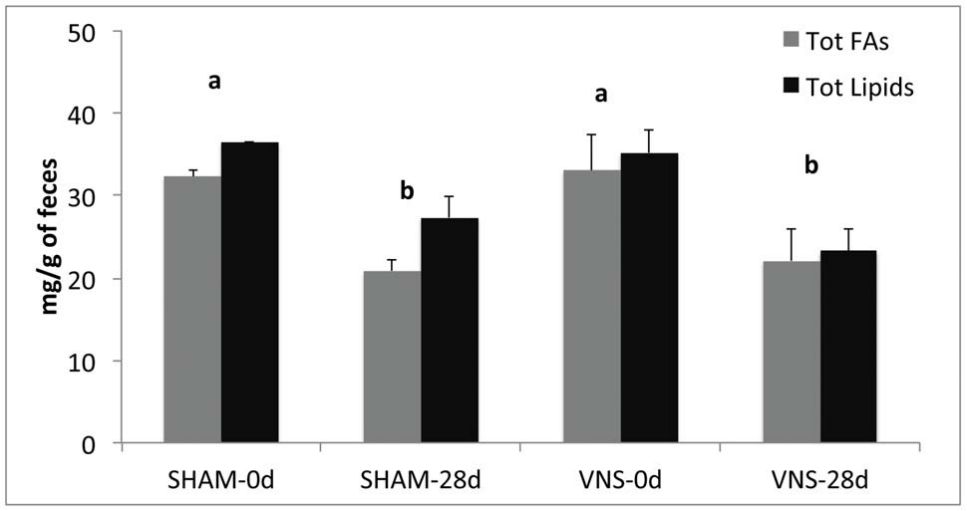
Total fatty acids (Tot FAs) and total lipids (Tot Lipids) in feces before (0d) and after treatment (28d) of sham-operated and subjected to VNS rats. Data are means ± SEM for four animals per group. Different letters denote significant differences (*P*<0.05).

### Animals

Male Sprague-Dawley rats (body weight, 250 to 300 g) were obtained from Charles River (Milan, Italy) and housed under standard laboratory conditions and left to acclimatize for 10 days prior surgery. They were anesthetized by intraperitoneal injection of Equithesin (1 g of sodium pentobarbital, 4.251 g of choral hydrate, 2.125 g of MgSO_4_, 12 ml of absolute ethanol, and 43.6 ml of propylene glycol, adjusted to a total volume of 100 ml with distilled water) at a dose of 1 ml per 300 g of body weight for the implantation of a VNS stimulator. A control group of animals was subjected to the same surgery with leads that were connected to a dummy pulse generator.

**Figure 4:**
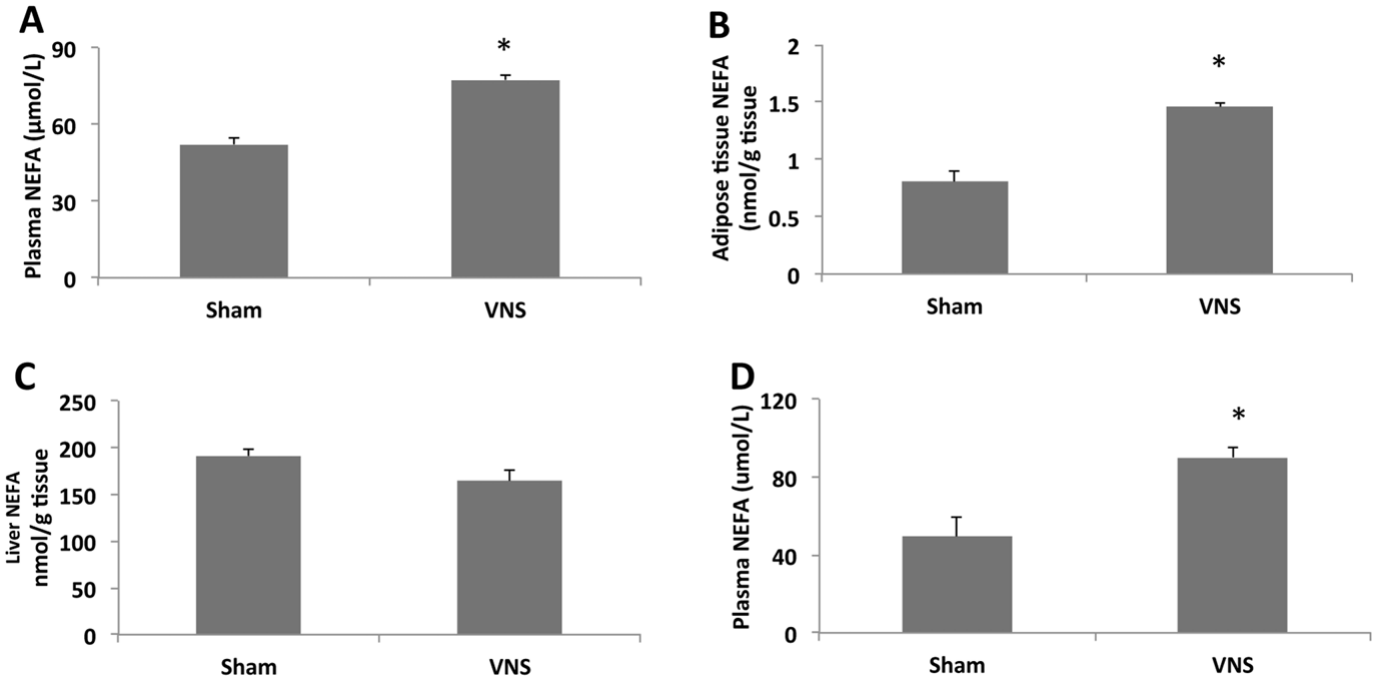
Effects of chronic VNS on mesenteric adipose tissue. (**A**) Weight of mesenteric adipose tissue in rats subjected to VNS (VNS), pair fed sham-operated (SHAM P-F) and ad libitum sham-operated (SHAM) rats for 4 weeks. Data are means ± SEM for thirteen animals per VNS, nine per SHAM, and four per pair fed SHAM. Different letters denote significant differences (*P*<0.05). (**B**) Abdominal adipose tissue of rats after SHAM or VNS treatment. The arrow indicates the reduced amount of perirenal fat in the VNS-treated rat.

Vagus nerve stimulator (model 103 DemiPulse Generator; Cyberonics, Houston, TX), comprising a pulse generator programmable by telemetry, was implanted in the upper left side of the back of each animal. A lead terminating in a double-coiled electrode was positioned on the cervical portion of the left vagus nerve. The stimulator was tested before implantation by serial connection to a dedicated palmtop.

**Figure 5:**
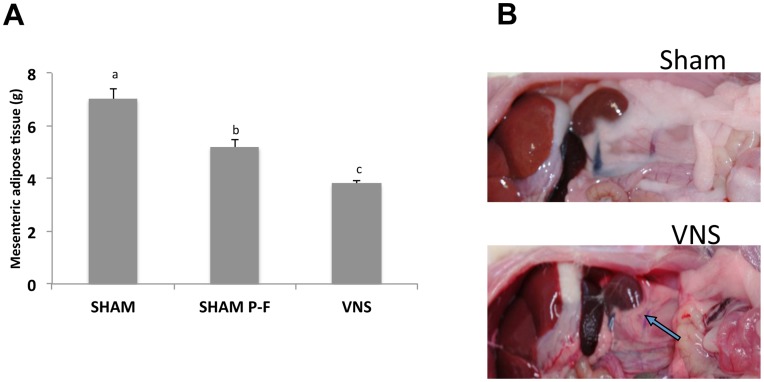
Effects of VNS on plasma and tissue levels of NEFA. NEFA levels were determined in plasma (**A**), mesenteric adipose tissue (**B**), and liver (**C**) of rats treated with VNS for 4 weeks or in the plasma of those subjected to acute VNS for 3 h (**D**). Data are means ± SEM for nine animals per group. *P<0.05 versus the corresponding value for sham-operated control rats (Scheffe’s test).

**Figure 6:**
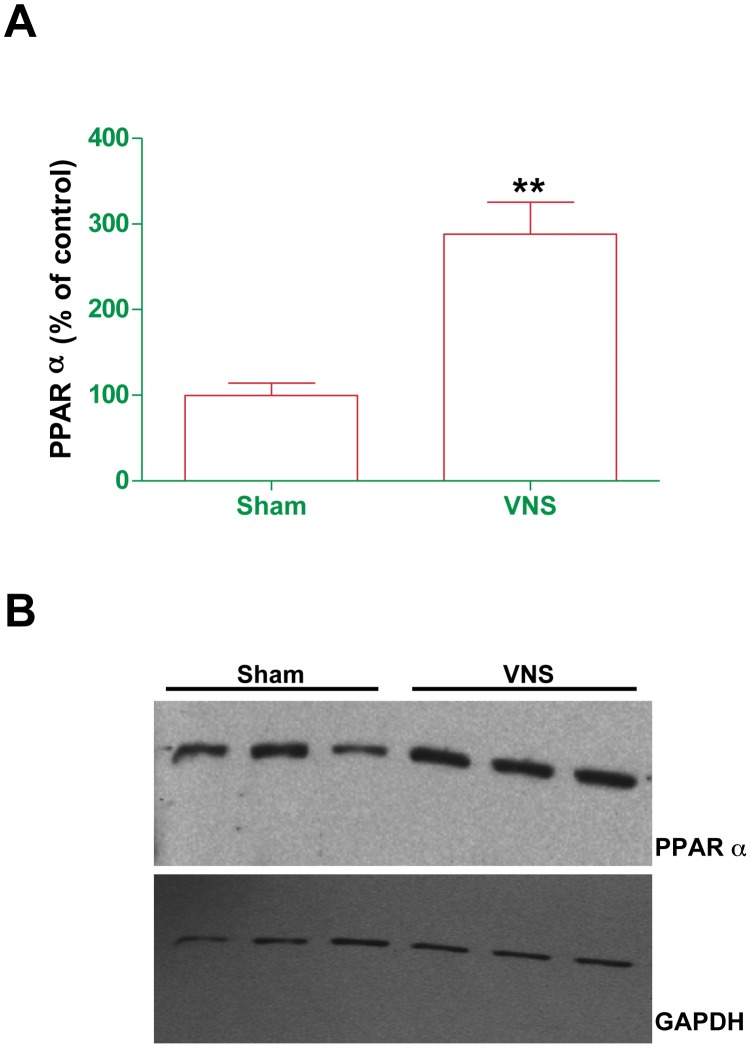
Effect of chronicVNS on the abundance of PPARα in the liver. Liver homogenate prepared from rats treated with VNS for 4 weeks or from sham-operated controls was subjected to immunoblot analysis with antibodies to PPARα. (A)The amount of PPARα was determined by densitometry, normalized by that of GAPDH, and expressed as a percentage of the value for sham-operated controls. Data are means ± SEM for eight VNS-treated and six sham-operated rats. **P<0.0001 versus the value for sham-operated controls (t test). (B) Representative immunoblot experiment.

Two days after surgery, the pulse generator was activated for chronic (4 weeks) treatment [9] or acute (3 h) stimulation [10].

The device was switched on at a current of 1.50 mA, a parameter which represents the average impulse used in epilepsy treatment. Such impulse was delivered at a current frequency of 30 Hz, pulse width 500 msec, cycling continuously with 30 sec on and 5 min off. Once set, these parameters were maintained throughout the duration of the study.

Food intake and body weight of individual animals housed in metabolic cages (Tecniplast, Varese, Italy) was monitored daily. Feed conversion efficiency was measured as the total food intake expressed in Kcal divided the weight gain for each animal during the treatment period. After stimulation, the animals were killed by decapitation, blood was collected from the aorta for isolation of plasma, and the liver, mesenteric adipose tissue, and hypothalamus were removed, weighed, and stored at −80°C until analysis.

**Figure 7:**
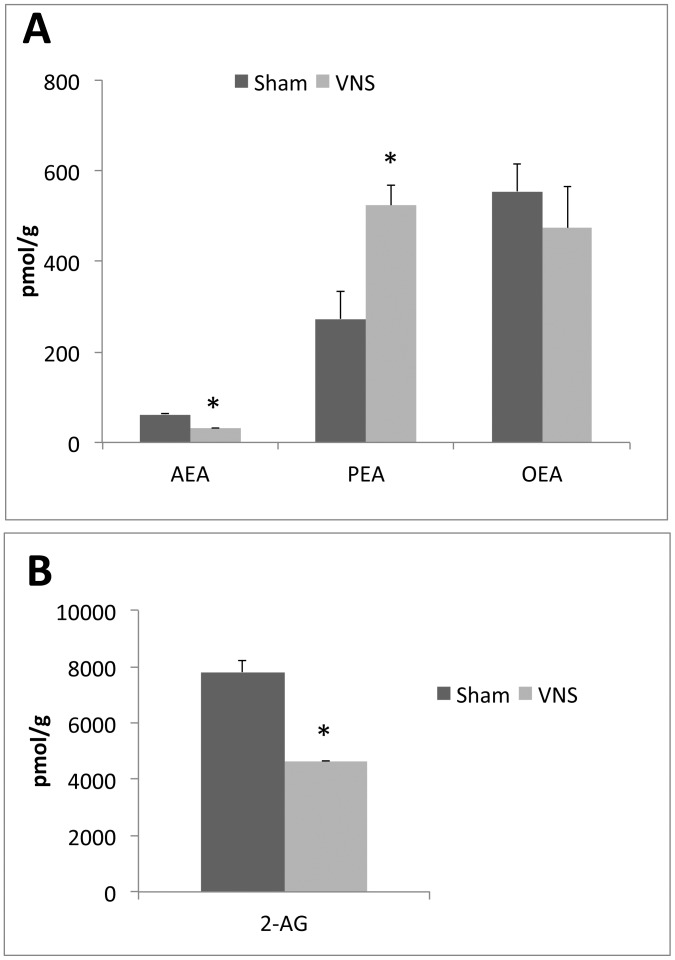
Effects of chronic VNS on the levels of endocannabinoids and the PPARα ligands PEA and OEA in mesenteric adipose tissue. The amounts of AEA, PEA, and OEA (**A**) and of 2-AG (**B**) in mesenteric adipose tissue were determined for rats subjected to VNS for 4 weeks and for sham-operated animals. Data are means ± SEM for four animals per group. *P<0.05 versus the corresponding value for sham-operated controls (Scheffe’s test).

**Figure 8:**
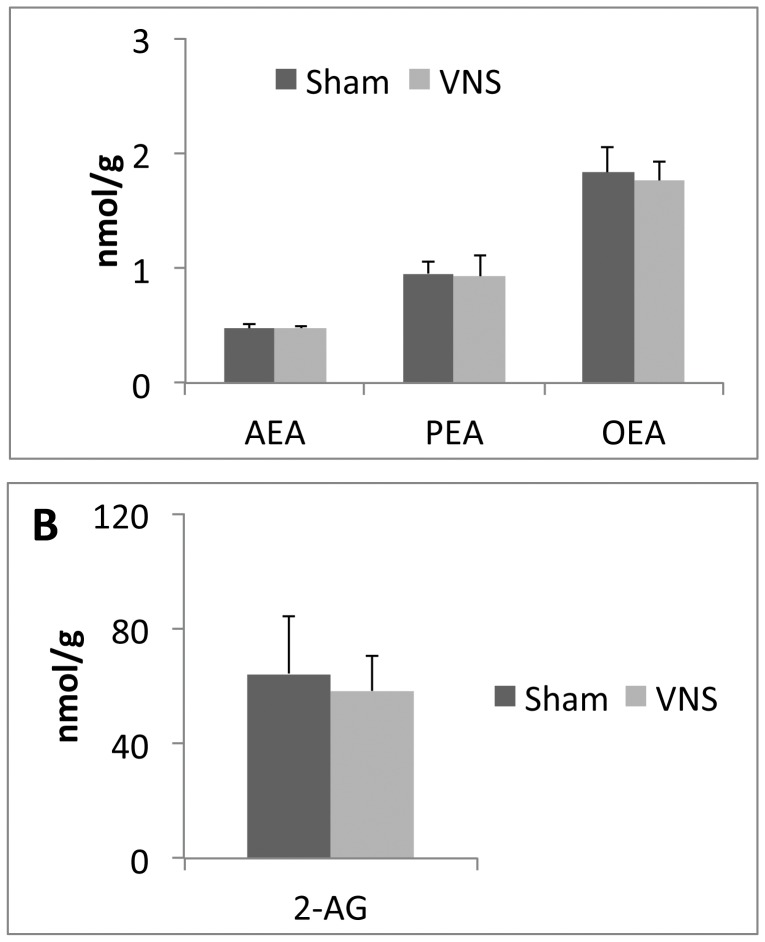
Effects of chronic VNS on the levels of endocannabinoids and the PPARα ligands PEA and OEA in the hypothalamus. The amounts of AEA, PEA, and OEA (**A**) and of 2-AG (**B**) in the hypothalamus were determined for rats subjected to VNS for 4 weeks and for sham-operated animals. Data are means ± SEM for four animals per group. No significant differences were detected between VNS-treated and control animals (Scheffe’s test).

**Figure 9:**
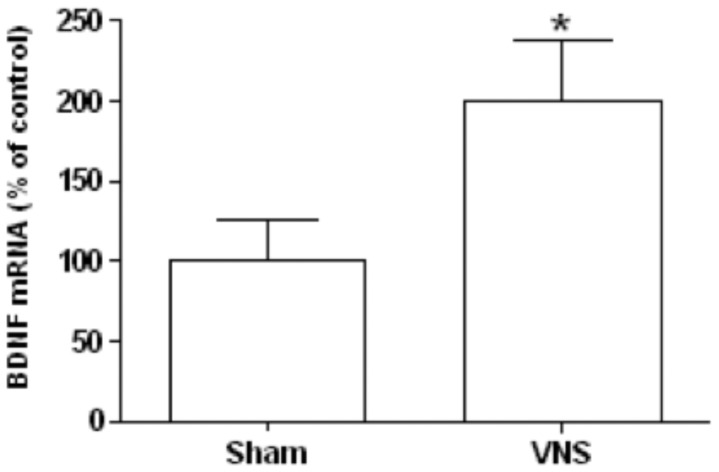
Effect of chronic VNS on the amount of BDNF mRNA in the hypothalamus. The abundance of BDNF mRNA in the hypothalamus was determined by RT and real-time PCR analysis for rats treated with VNS for 4 weeks and for sham-operated controls. Data were normalized by the amount of βIV-tubulina mRNA, are expressed as a percentage of the value for sham-operated controls, and are means ± SEM for nine animals per group. *P<0.05 versus sham-operated controls (Scheffe’s test).

### Pair Feeding Experiment

An ancillary experiment aimed at assessing on whether decrease of body weight and adipose mesenteric tissue is exclusively related to changes in food intake, was carried out using a pair-feeding paradigm.

Two naïve, 4 SHAM and 4 VNS rats were used in this experiment. During the four weeks of VNS stimulation, each SHAM operated rat was pair-fed by providing the same amount of food, consumed on the previous day, by the matched VNS rat. The two-naïve rats were used as an internal control to monitor the expected reduction of food intake induced by VNS.

### Measurement of Lipids, NEFA, and Endocannabinoids

Total lipid was extracted by the method of Folch et al. [24]. Separation of major lipid classes was carried out as described in [25]. Briefly, aliquots of the organic phase containing about 5 mg of total lipid in 0.5 ml of chloroform were placed on an aminopropyl bond large reservoir capacity column (Chrompack International BV, Middleburg, The Netherlands) for solid phase extraction. Each lipid class was eluted using different solvents: 10 ml of chloroform–isopropanol (2∶1, v/v) for triacylglycerol fraction; 5.5 ml of acetic acid–diethyl ether (2∶98, v/v) for free fatty acid (FFA); 20 ml of methanol for PLs). Each fraction was collected and the solvent evaporated. With the exception of the FFA fraction, the other lipid fractions were mildly saponified as previously described [26] in order to obtain free fatty acids. Free fatty acids were methylated and analyzed by means of a gas chromatograph (Agilent, Model 6890, Palo Alto, CA) as described in [27].

Total lipids were quantified as described in [28].

The lipid-containing organic phase was dried down, weighed and pre-purified by open bed chromatography on silica gel. Fraction was obtained by eluting the column with 90∶10 (v/v) chloroform/methanol. This fraction was used for AEA, 2-AG, PEA and OEA quantification by liquid chromatography-atmospheric pressure chemical ionization-mass spectrometry (LC-APCI-MS), and using selected ion monitoring at M +1 values for the four compounds and their deuterated homologues, as described in [29].

### Isolation of RNA and Measurement of BDNF mRNA

Total RNA was isolated from the hypothalamus by the guanidine isothiocyanate method as previously described [10]. Portions (1 µg) of the RNA were subjected to RT with the use of an iScript cDNA synthesis kit (Bio-Rad, Milan, Italy), and the resulting cDNA was subjected to real-time PCR analysis with primers specific for BDNF [30]. PCR amplification was performed with the use of iQ SYBR Green Supermix (Bio-Rad) and a C1000 thermal cycler (Bio-Rad) for 30 cycles of incubation at 94°C for 60 s, 57°C for 60 s, and 72°C for 60 s followed by incubation at 70°C for 10 min. βIV-Tubulin mRNA was quantified as an internal control for normalization of BDNF mRNA [30]. PCR products were also subjected to electrophoresis on a 2% agarose gel and stained with ethidium bromide to verify amplification size.

### Immunoblot Analysis

The liver was homogenized in 10 volumes (1 g/10 ml) of a solution containing 10 mM Tris-HCl (pH 7.4), 0.32 M sucrose, 5 mM EDTA, 0.1 mM phenylmethylsulfonyl fluoride, 1 mM benzamidine, aprotinin (1 ml/l), and bacitracin (200 µg/ml), and the homogenate was centrifuged at 1000×*g* for 20 min at 4°C. The resulting pellet was resuspended in homogenization buffer and centrifuged at 10,000×g for 10 min at 4°C. The new pellet was resuspended in homogenization buffer and stored at −80°C until analysis. Portions (40 µg of protein) of the thawed samples were fractionated by SDS-PAGE on a 10% gel, the separated proteins were transferred to a polyvinylidene difluoride membrane, and the membrane was exposed for 1 h at room temperature to 5% dried skim milk in PBST (PBS containing 0.05% Tween-20, pH 7.2) before incubation first overnight at 4°C with rabbit polyclonal antibodies to PPARα (Santa Cruz Biotechnology, Santa Cruz, CA) and then for 1 h at room temperature with HRP-conjugated antibodies to rabbit (Amersham-Pharmacia Biotech, Arlington Heights, IL). The membrane was washed three times with PBST for 10 min between each step. Immune complexes were finally detected with the use of ECL reagents (Amersham-Pharmacia Biotech). The membrane was subsequently stripped and re-probed with mouse monoclonal antibodies to glyceraldehyde-3-phosphate dehydrogenase (GAPDH) followed by HRP-conjugated goat polyclonal antibodies to mouse IgG (Amersham-Pharmacia Biotech). The intensity of the PPARα band was determined by densitometry with the use of a Bio-Rad GS-700 Imaging Densitometer and was normalized by that of the GAPDH band (internal control).

### Statistical Analysis

Quantitative data are presented as means ± SEM and were analyzed by one-way ANOVA followed by Scheffe’s test or by the *t* test as indicated. A *P* value of <0.05 was considered statistically significant.

## Results

VNS for 4 weeks in male rats resulted in significant decreases of about 12% of food intake (1972.8±47.6 vs 1739±33.5 as KCal intake in 4 weeks treatment) (figure 1A) and ∼25% in weight gain (75.1±1.1 vs 56.6±2.4 g body weight in 4 weeks treatment) (figure 1B) compared with sham-operated control rats. Feed conversion efficiency (FCE) was thus significantly decreased by chronic VNS, with VNS-treated rats requiring an intake of ∼45 kcal to gain 1 g of weight compared with ∼30 kcal/g for controls (figure 1C). The daily body weight measurement showed a steady body weight gain reduction in VNS rats from 1 week-treatment, with an average of 20 g lower than sham-treated rats (figure 2A). Daily food intake was slightly lower almost every day with an average of dietary intake of 30.3±2.6 g/d in sham-operated rats and 26.4±1.7 g/d in VNS treated rats (figure 2B). To establish whether the decrease in FCE was the result of reduced fat absorption due to increased gastrointestinal tract motility, we measured the amount of fat but also total fatty acids in feces because a small fraction of fat in the feces consists in biliary acids and cholesterol which do not influence calorie absorption. As depicted in figure 3, both total lipid and fatty acids content of feces before and after sham or VNS treatment were statistically different, while the values after treatment for VNS-treated rats did not differ significantly from those for controls, suggesting that a difference in fat absorption was not responsible for the difference in FCE.

In the pair-fed experiment, the reduction in body weight between VNS and pair-fed sham (mean±SEM, 7.48% ±0.4), even though significant, was less evident with respect to ad libitum sham. On the other hand, the weight of mesenteric adipose tissue in rats treated with VNS for 4 weeks was reduced by ∼45% compared with that in sham-operated animals and about 26% when compared to pair fed sham (figure 4), demonstrating that after 4 weeks of treatment the reduction in food intake induced in pair-fed SHAM operated rats did not prevent to observe the marked reduction in mesenteric fat accumulation in VNS rats. Moreover, we calculate the average amount of food consumed, expressed in grams per kilogram of body weight, during the 4-week treatment. We observed that the reduction in food intake in VNS rats compared to the amount consumed by at libitum fed naïve rats was 14.75% ±2.7 (mean±SEM), similar to the reduction observed in the first experiment (mean±SEM, 14.23% ±2.9) comparing VNS rats with at libitum fed SHAM rats. On the contrary, the value calculated for pair-fed SHAM rats compared to at libitum fed naïve rats was only 4.41±3.1 (mean±SEM) due to the expected reduction in body weight induced by caloric restriction in these animals.

The reduction in mesenteric fat mass induced by VNS was accompanied by significant increases of ∼50 and ∼80% in the NEFA content of plasma (figure 5A) and mesenteric adipose tissue (figure 5B), respectively, with no corresponding effect on that of the liver (figure 5C). Furthermore, triacylglycerol accumulation in adipose tissue and liver did not differ significantly between VNS and sham treated rats (data not shown). To clarify whether these changes were directly related to VNS or were due to nutrient displacement, we subjected rats to acute VNS for 3 h and then measured the NEFA concentration in plasma. Such acute VNS also induced a significant increase in plasma NEFA level (figure 5D). These results thus suggested that VNS may increase lipolysis in adipose tissue and fatty acid oxidation in the liver. This notion was further supported by the observation that chronic VNS increased the hepatic abundance of PPARα (figure 6), a transcription factor that regulates the expression of various genes related to fatty acid oxidation and uptake.

To gain more insight into the molecular mediators of the observed effects of VNS, we measured the concentrations of the endocannabinoids AEA and 2-AG as well as of the metabolically related PPARα ligands PEA and OEA in both adipose tissue and the hypothalamus as well as the expression of the gene for the anorexic neurotrophin BDNF in the hypothalamus. The amounts of both AEA and 2-AG in mesenteric adipose tissue were markedly decreased by chronic VNS, whereas the amount of PEA was significantly increased and that of OEA was unaffected by such treatment (figure 7). The amounts of these four compounds in the hypothalamus were not affected by chronic VNS (figure 8). Finally, the abundance of BDNF mRNA was significantly increased in the hypothalamus of VNS-treated rats compared with that in sham-operated control animals (figure 9).

## Discussion

The effects of the various possible combinations of programmable VNS options other than those established in antiepileptic protocols (current intensity, on-off operating time, pulse duration) have not been fully investigated, although recent evidence suggests that different settings obtained by telemetric modification of the battery delivering VNS may result in clinical outcomes other than anticonvulsant effects, such as reduction of cerebellar tremor and improvement of dysphagia in subjects affected by multiple sclerosis [31], [32]. Weight loss has also been observed in some epileptic patients treated with VNS [2], [4], [6], but the effect of this therapy on body weight has remained unclear [5]. In general, peripheral vagal stimulation may induce weight loss by engaging vagal afferents from the gastrointestinal tract that mediate satiety [33], given that the intestinal hormone cholecystokinin (CCK) inhibits food intake via stimulation of these afferent neurons. However, other possible effects of VNS on gastrointestinal tract motility are unlikely to account for the changes in body composition observed in the present study. In fact, since stimulation was applied to the vagus nerve at the cervical level where ∼80% of the fibers in the truncal, compact part of the vagus nerve are afferent, with only the remaining 20% being efferent, we mainly stimulated the central rather than peripheral nervous system. Furthermore, in support of this consideration, while a stimulatory effect on gastrointestinal tract motility would be expected to reduce absorption and thereby increase the amount of fat in feces, we failed to detect such an increase. Nevertheless, at present a possible contribution of the efferent nerves cannot be excluded.

We found that VNS for 4 weeks induced an ∼33% decrease in FCE, an ∼25% reduction in weight gain, and an ∼45% decrease in the amount of mesenteric adipose tissue. These effects were accompanied by ∼50 and ∼80% increases in the NEFA levels of plasma and mesenteric adipose tissue, respectively. In addition, VNS treatment for only 3 h also increased the plasma NEFA concentration, ruling out long term food intake or absorption related mechanisms. These data indicate that during VNS there could be a mobilization of fatty acids from adipose tissue and their transport via the plasma for storage or metabolism in other peripheral tissues such as the liver. However, we neither detected fat accumulation nor an increased NEFA content in the liver of rats subjected to chronic VNS, suggesting that the increased fat mobilization from adipose tissue is associated with increased fatty acid oxidation in the liver. This hypothesis was supported by the marked increases both in the hepatic abundance of PPARα, a key transcription factor related to fatty acid oxidation, and in the amount of PEA, one of the most potent endogenous ligands of PPARα [34], [35], in adipose tissue. NEFA may thus be mobilized from adipose tissue and transported to the liver, where activated PPARα may induce the transcription of genes involved in NEFA oxidation. Moreover, as far as we are aware, this is the first demonstration that a nonpharmacological and nonnutritional intervention is able to up-regulate the expression of PPARα and the production of endocannabinoids in peripheric tissues.

Down-regulation of the endocannabinoid system has been demonstrated following the vagus nerve after stimulation [36]. It is possible that a down-regulated endocannabinoid tone in the periphery may explain the reduced fat mass observed in VNS-treated rats in the present study. Indeed, blockade of peripheral cannabinoid receptors has been shown to reduce body fat [37]. In addition, the decrease in the amounts of endocannabinoids along with the increase in that of PEA observed in adipose tissue of VNS-treated rats reported in the present study, may contribute to explain the previously demonstrated anti-inflammatory effect of VNS [38].

Thus, the overall action of VNS in our experimental model may be due to the combination of distinct and complementary mechanisms with multiple players involved, that among the others mentioned above may also include BDNF. Accordingly, we previously showed that VNS induced a long-lasting increase in BDNF expression in various regions of the rat brain [9], [10]. We have now shown that VNS increases expression of the BDNF gene in the hypothalamus, a key brain region in the control of body weight [39]. Indeed, among its many activities, BDNF contributes to the hypothalamic melanocortin-driven pathway that may by involved in the control of body weight, food intake and possibly energy expenditure influencing FCE and the partitioning of fuel stores into fat [39].

In support of a role in energy expenditure, Wang et al. [40], [41] found that selective BDNF administration into the ventral median hypothalamus (VMH) or paraventricular nucleus (PVN) resulted in elevated basal metabolic rate.

Interestingly VMH lesions trigger a reduction of sympathetic nervous system activity in several organs including white adipose tissue [42], leading to decreased catecholamine turnover [43].

Although still speculative, our hypothesis suggests that VNS might modulate food intake and energy expenditure by inducing BDNF in the hypothalamus leading to an increased catecholamine tone in adipose tissue, supported by the increased lipolysis. In addition, VNS impinges on catecolaminergic projection to the hypothalamus by activation of relay projections through A1 noradrenergic cell group of the caudal ventrolateral medulla [44], though possible sympathetic effects can be related to VNS impingement upon the locus coeruleus via nucleus of the solitary tract [45]. In addition norepinephrine contents have been found increased by VNS in prefrontal and lymbic areas [10].

Catecholamine tone is also known to decrease visceral adipose tissue greater than subcutaneous adipose tissue [46]. In the present study VNS treatment reduced mesenteric fat by 46%, while in the pair fed rats this reduction was only of 26%.

Thus, the pair-feeding experiment suggested, as expected, that food intake restriction induced a decrease in body weight and mesenteric fat also in sham animals and that VNS treatment has an effect on energy expenditure by inducing a decrease in mesenteric fat that seems to be independent by food intake. The reduction of body weight by VNS when compared to pair-fed sham was less marked than the reduction of mesenteric fat, suggesting that VNS activity is more targeted toward adipose tissue. However, whether the reduction of weight gain is entirely due to a decreased adipose depots or also lean mass is involved, was not assessed in the present study. The daily body measurement curve showed that the decrease started during the second week with a stable growth curve thereafter. This might suggest a decreased fat accumulation in the second week and a stable weight gain thereafter.

The present study reveals the potential role of a concerted action of BDNF in the hypothalamus, the endocannabinoid system in adipose tissue, and PPARα in the liver, in promoting weight loss following VNS. Additional studies are needed to investigate in more details the molecular pathways affected by VNS in CNS and peripheral tissues in order to determine whether the decrease of body fat might be mediated in humans by the same mechanisms operative in experimental models. Moreover, these data may provide both a new avenue for exploring the mechanisms underlying severe weight gain and as well as a potential safe and low-level surgical approach to obesity treatment.
